# Age at menarche and prevention of hypertension through lifestyle in young Chinese adult women: result from project ELEFANT

**DOI:** 10.1186/s12905-018-0677-y

**Published:** 2018-11-09

**Authors:** Liqiong Guo, Cheng Peng, Hui Xu, Ander Wilson, Peng-hui Li, Hao Wang, Hongbin Liu, Lilin Shen, Xi Chen, Xiuying Qi, Nai-jun Tang, Timothy M. Barrow, Hyang-Min Byun

**Affiliations:** 1 Department of Occupational & Environmental Health, School of Public Health, Tianjin Medical University, 22nd Qixiangtai Road, Heping District, Tianjin, 300070 China; 2 Department of Environmental Health, Harvard T.H. Chan School of Public Health, Boston, MA USA; 3 Department of Epidemiology and Statistics, School of Public Health, Tianjin Medical University, Tianjin, China; 4 Department of Statistics, Colorado State University, Fort Collins, CO USA; 5 School of Environmental Science and Safety Engineering, Tianjin University of Technology, Tianjin, China; 6 Tianjin Research Institute for Family Planning, Tianjin, China; 7 Faculty of Health Sciences and Wellbeing, University of Sunderland, Sunderland, UK; 8 Human Nutrition Research Centre, Institute of Cellular Medicine, Newcastle University, Newcastle upon Tyne, UK

**Keywords:** Menarche, Hypertension, Lifestyle

## Abstract

**Background:**

Early and late age at menarche are associated with risk of hypertension, but little is known whether modifiable lifestyle can reduce this risk.

**Methods:**

Our study leverages 60,135 healthy young Chinese women from the Environmental and LifEstyle FActors iN metabolic health throughout life-course Trajectories (ELEFANT) study. Menarche age and lifestyle factors were assessed by self-reported questionnaires and hypertension was diagnosed by physicians. We estimated the odds ratios (ORs) of hypertension associated with menarche age using multivariable logistic regression. We further investigated whether modifiable lifestyles (body mass index, BMI; psychological stress; passive smoking; and imbalanced diet) increased risk in joint analyses.

**Results:**

The association between age at menarche and hypertension was U-shaped, with age ≤ 12 at menarche giving the highest OR (1.46, 95% confidence interval [CI], 1.27–1.69) and ≥ 16 the second highest (OR = 1.36, 95% CI = 1.15–1.62). Simultaneous analysis of lifestyle risk factors and age of menarche showed that having one or more modifiable risk factors increased the menarche age-hypertension association. The risk of hypertension among participants with menarche age ≤ 12 decreased from OR 13.21 (95% CI = 5.17–29.36) with four high-risk lifestyle factors to 12.36 (95% CI = 9.51–16.05) with three high-risk factors, 5.24 (95% CI = 4.11–6.69) with two, and 2.76 (95% CI = 2.09–3.60) with one, in comparison to individuals with no high-risk lifestyle factors and menarche age 14.

**Conclusions:**

Our results suggest that modification of lifestyle, including maintenance of normal weight and a balanced diet, are associated with substantially reduce the risk of hypertension in high-risk individuals.

**Plain English summary:**

Early and late age at menarche are risk factors for the development of hypertension in Western populations, and there is limited evidence that this is also true of Chinese populations. Targeted prevention of hypertension in vulnerable populations would be highly beneficial in efforts to reduce the incidence of cardiovascular disease, but it is not currently known whether lifestyle intervention could reduce hypertension risk.

In this study, we analysed the risk of hypertension by age at menarche and four modifiable lifestyle factors (BMI, diet, psychological stress, and smoking tobacco) in a cohort of 60,135 young adult Chinese women (mean age 29).

We identified that early and late age at menarche are associated with increased risk of hypertension in young Chinese women. There was joint effects between age at menarche and lifestyles on hypertension only participants with age at menarche ≤12 and being overweight or obese. Modification of lifestyle, including maintenance of normal weight and a balanced diet, can substantially reduce the risk of hypertension in high-risk individuals.

In conclusion, our study has revealed that early and late menarche age are associated with the development of hypertension in young Chinese women, and that this risk is modified by healthy lifestyle traits.

**Electronic supplementary material:**

The online version of this article (10.1186/s12905-018-0677-y) contains supplementary material, which is available to authorized users.

## Background

Age at menarche is determined by genetic profile, hormonal levels, and external factors such as exposure to environmental chemicals, diet, physical activity, and smoking [1–4]. Early and late menarche age are known to be risk factors associated with adult onset diseases, including ischaemic heart disease, and hypertension, and may in part explain sex differences in the risk of cardiovascular disease (CVD) [5–8]. Although hypertension is most prevalent in elderly populations and displays increased risk with ageing, there remains a considerable number of hypertension cases among young adults independent of familial history of hypertension [9, 10]. The early prevention of hypertension in vulnerable individuals, such as young adult women with early or late age at menarche, is cardinal. Healthy lifestyle has been suggested as a long-term approach for the prevention of diseases including hypertension [11], and may ameliorate the increased risk associated with early or late age at menarche.

In this study, we assessed the risk of hypertension by menarche age and by level of four modifiable lifestyle factors in young adult Chinese women. The four modifiable factors were: body mass index (BMI); psychological stress; smoking status; and imbalanced diet. By estimating the synergistic association of lifestyle factors and age of menarche on hypertension risk, we demonstrate the high-impact potential for intervention to reduce incidence in high-risk individuals.

## Methods

### The ELEFANT participants

Project Environmental and LifEstyle FActors iN metabolic health throughout life-course Trajectories (ELEFANT) is a population study comprising three age-based cohorts: Baby ELEFANT (mean age 0); Young ELEFANT (mean age 29); and Elderly ELEFANT (mean age 69) [12]. All participants resided in Tianjin city, China (area: 11760 km^2^, population: 156,200,000 in 2016) at the time of recruitment. In this study, we utilised the Young ELEFANT cohort, which is a cross-sectional study for which data collection began in 2014. The total number of participants within Young ELEFANT is 124,286 (50% male). The participants are living in Tianjin city who visited clinics in Tianjin between 2014 and 2015 for regular check-ups. Basic demographic and clinical characteristics were collected through questionnaires, which measured socioeconomic and lifestyle data such as education level, occupation, smoking status, drinking behaviour, psychological stress, and dietary habits during the period 2014 to 2015. Reproductive characteristics (age at menarche, menstrual cycle length, menstrual bleeding duration, and dysmenorrhea), personal history of disease, familial history of disease, and medication use were also collected (Table 1). The protocol of this study was approved by the Institutional Review Board of the Tianjin Medical University and the procedures followed were in accordance with institutional guidelines. Participants gave written informed consent prior to participation in the study.

**Table 1:** Basic characteristics of the study population by age at menarche

	Age at menarche					
Variables	Total (*n* = 60,135)	≤12	13	14	15	≥16
		(*n* = 9347)	(*n* = 13,476)	(*n* = 23,184)	(*n* = 9017)	(*n* = 5108)
Age at enrolment, mean (SD)	29 (4)	30 (4)	30 (40)	29 (4)	29 (4)	30 (4)
BMI (kg/m^2^), mean (SD)	23 (4)	23 (4)	23 (4)	22 (4)	22 (4)	22 (4)
Overweight (BMI 24–28), *n* (%)	11,613 (19)	2060 (18)	2652 (23)	4319 (37)	1621 (14)	961 (8)
Obese (BMI ≥ 28), *n* (%)	4244 (7)	894 (21)	973 (23)	1474 (35)	580 (14)	323 (8)
SBP (mmHg), *n* (%)	112 (10)	112 (11)	112 (10)	112 (10)	112 (10)	112 (10)
DBP (mmHg), *n* (%)	72 (8)	72 (9)	72 (8)	72 (8)	72 (8)	72 (8)
Alcohol drinker, *n* (%)	4237 (7)	1388 (33)	1022 (24)	988 (23)	450 (11)	389 (9)
Active smoker, *n* (%)	774 (1)	241 (31)	182 (24)	167 (22)	101 (13)	83 (11)
Passive smoking, *n* (%)	14,892 (25)	4103 (28)	3622 (24)	3683 (25)	1926 (13)	1558 (10)
Imbalanced diet, *n* (%)	1350 (2)	274 (20)	327 (24)	359 (27)	181 (13)	209 (15)
Education (years)						
≤ 9, *n* (%)	22,537 (37)	1466 (7)	4698 (21)	10,159 (45)	4122 (18)	2092 (9)
10~ 15, *n* (%)	11,372 (19)	1507 (13)	2518 (22)	4540 (40)	1696 (15)	1111 (10)
≥ 16, *n* (%)	26,181 (44)	6362 (24)	6250 (24)	8473 (32)	3194 (12)	1902 (7)
Occupation						
Manual work, *n* (%)	43,286 (73)	4454 (10)	9250 (21)	18,632 (43)	7149 (17)	3801 (9)
Non-manual work, *n* (%)	15,178 (26)	4294 (28)	3841 (25)	4170 (27)	1709 (11)	1164 (8)
Unemployed, *n* (%)	883 (2)	298 (34)	200 (23)	213 (24)	94 (11)	78 (9)
Region						
Rural, *n* (%)	39,233 (65)	3242 (8)	8409 (21)	17,356 (44)	6662 (17)	3564 (9)
Urban, *n* (%)	20,911 (35)	6105 (29)	5076 (24)	5830 (28)	2356 (11)	1544 (7)
Psychological stress, *n* (%)	14,306 (24)	4306 (30)	3560 (25)	3505 (25)	1726 (12)	1209 (8)
Parity						
None, *n* (%)	28,325 (47)	5920 (21)	6600 (23)	10,210 (36)	3664 (13)	1931 (7)
≥ 1, *n* (%)	31,810 (53)	3427 (11)	6876 (22)	12,976 (41)	5354 (17)	3177 (10)
Oral contraceptive use, *n* (%)	417 (1)	115 (28)	113 (27)	102 (24)	44 (11)	43 (10)
Family history of hypertension, *n* (%)	95 (0)	28 (29)	23 (24)	28 (29)	10 (11)	6 (6)
Diabetes, *n* (%)	809 (1)	157 (19)	187 (23)	274 (34)	115 (14)	76 (9)
Hypertension, *n* (%)	1798 (3)	412 (23)	414 (23)	541 (30)	241 (13)	190 (11)

*BMI* body mass index, *SBP* systolic blood pressure, *DBP* diastolic blood pressure, oral contraceptive use was defined as current or ever use

### Sampling process and eligibility criteria

**Fig. 1:**
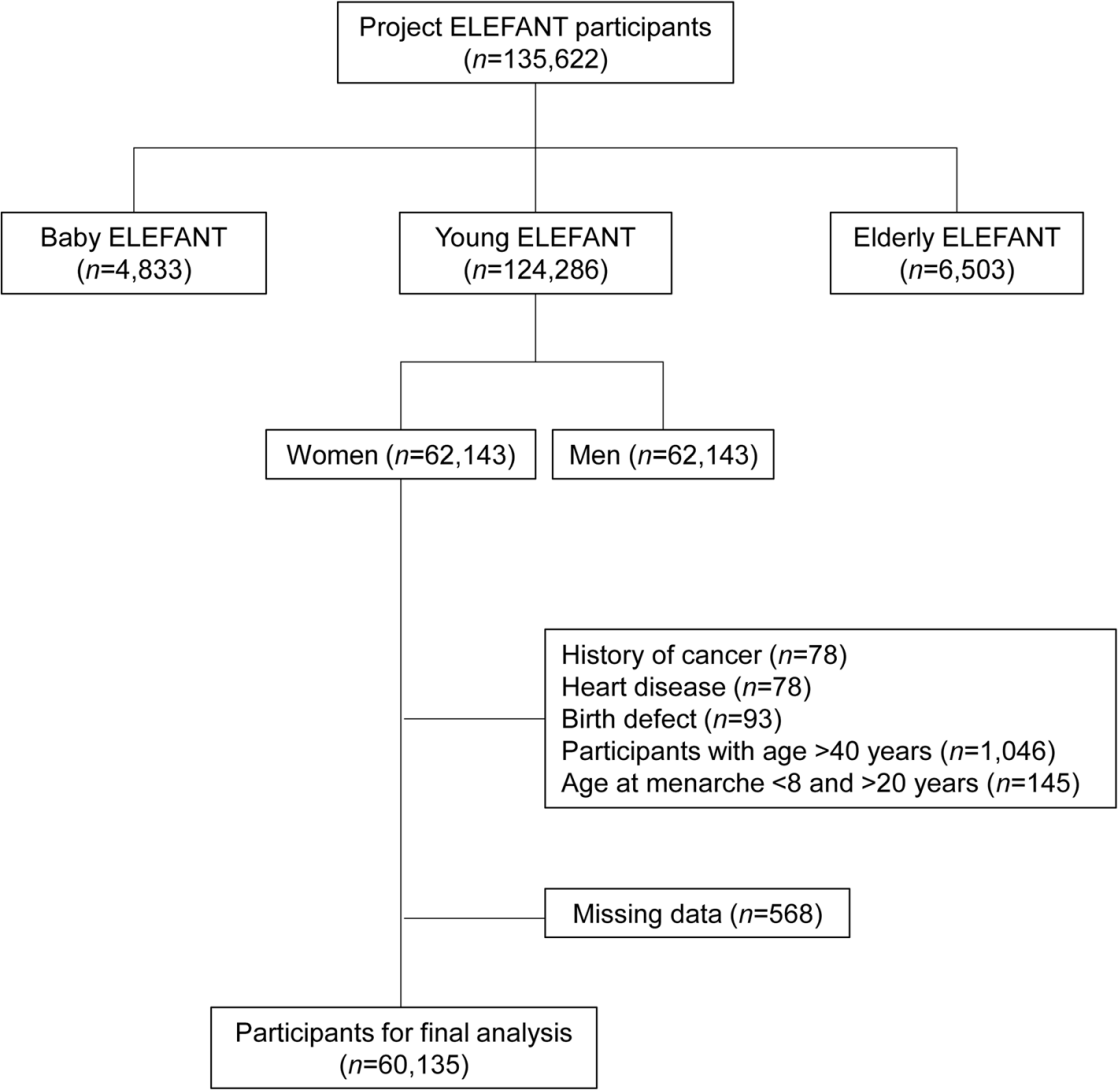
Participant selection flow chart for the study

The selection process for female participants within the Young ELEFANT cohort is described in Fig. 1*.* We excluded participants with history of cancer (*n* = 78), heart disease (*n* = 78), birth defect (*n =* 93), participants aged > 40 years (*n =* 1046), and those with age at menarche of < 8 years (*n* = 5) and > 20 years (*n =* 140). We also excluded participants with missing data (*n =* 568). Following these exclusions, 60,135 participants aged 24–40 were included in the final analysis for this study.

### Ascertainment of questionnaire data

Self-reported menarche age from questionnaire data for each participant were categorised as ≤12, 13, 14, 15, and ≥ 16 years. Median age of menarche in China is 14-year-old and was subsequently used as the reference age in our analysis [13].

Psychological stress was estimated through structured questionnaires to determine whether participants experienced work-related stress, social stress, or financial stress. The Occupational Stress Indicator (OSI) [14] and the Perceived Stress Scale (PSS-10) [15] were used. The PSS-10 is a self-report instrument which measures the perception of stress and ability to cope, such as how unpredictable, uncontrollable and overloaded the individual perceives their life to be. The OSI assess occupational satisfaction and stress by measurement of 40 items regarding stressors and job satisfaction on a 6-point Likert-type scale. The questionnaires captured the degree of stress from “none”, “low”, and “high” and we dichotomised as yes (high or low) or no (none) for the analysis. Both of questionnaires were validated and widely utilised in China and as well as across the world. The questionnaires were built to identify the psychological stress of the participants in the preceding 10 years.

Participants were categorised by cigarette smoking behaviours as non-smokers (including ex-smokers) and current smokers, and also by passive smoking status into non-passive smokers and passive smokers. Alcohol drinking behaviours were categorised into non-drinkers and current drinkers. Imbalanced diet was defined as that which predominantly or wholly involved consumption of one of vegetables, eggs or meats.

### Ascertainment of clinical measurements

Blood pressure (BP) was measured twice in the upper left arm after 5–10 min of rest in a seated position using an automated device (HBP-9021 J, Omron, Japan). The mean of these two measurements was used for further analysis. Hypertension was classified by physicians according to history of diagnosed hypertension, use of blood pressure medication or measured systolic blood pressure (SBP) ≥140 mmHg and/or diastolic blood pressure (DBP) ≥90 mmHg according to criterion of the JNC 7 (Seventh Report of the Joint National Committee on Prevention, Detection, Evaluation, and Treatment of High Blood Pressure) [9].

BMI was calculated as weight divided by height squared (kg/m^2^), measured at local hospitals. Based on the Department of Disease Control Ministry of Health People’s Republic of China (PRC), we considered participants with a BMI below 24 as normal weight, between 24 and 28 as overweight, and a BMI higher than 28 as obese [16].

### Statistical analysis

Multivariable logistic regression analyses were performed to estimate odds ratios (ORs) and 95% confidence intervals (CIs) for hypertension in relation to age at menarche (categorised into ≤12, 13, 14, 15, ≥16 years, with 14 years of age as the reference group) and unhealthy lifestyles (BMI, psychological stress, smoking status, passive smoking status, and imbalanced diet) separately. To determine whether potential confounders could affect the ORs for hypertension by age at menarche, we used a multivariable logistic regression model adjusting for age at enrolment, smoking status, passive smoking status, drinking status, education, occupation, region, psychological stress, diabetes, family history of hypertension and other reproductive characteristics including parity, and oral contraceptive use. The separate multivariable logistic models for the relation between hypertension and unhealthy lifestyles were adjusted for age at enrolment, education, occupation, region, diabetes, family history of hypertension, parity, and oral contraceptive use and other unhealthy lifestyles.

The combined association of age at menarche and any one of the unhealthy lifestyles was assessed by including categorical variables for both hypertension and the lifestyle factor and including the interaction between the two categorical variables into the regression model. Multivariable logistic regressions were performed to analyse the associations between those combined associations and hypertension adjusting for age at enrolment, education, occupation, region, diabetes, family history of hypertension, parity, and oral contraceptive use and other unhealthy lifestyles. Multiplicative interactions between age at menarche and traditional risk factor categories was examined by inputting the product interaction terms in the logistic regression model adjusting for the above potential confounders using the likelihood ratio test.

To examine the joint effect of multiple of modifiable risk factors and age at menarche on hypertension, we categorised the each modifiable risk factor into two groups, low or high, and created a composite variable specified as the number of high risk factors an individual had. For example, the lowest risk group was defined as those with uniformly healthy lifestyles - none of these risk factors. The highest risk group was those who exclusively had the unhealthy lifestyle characteristics and were thus categorised as high risk for all four modifiable risk factors. Multivariable logistic regressions were performed to analyse the joint effects of age at menarche and combinations of modifiable risk factors adjusting for age at enrolment, education, occupation, region, diabetes, family history of hypertension, parity, and oral contraceptive use. Multiplicative interactions between age at menarche and combinations of modifiable risk factors were also examined by inputting the product interaction terms in the logistic regression model adjusting for the above potential confounders using the likelihood ratio test. Bivariate and multivariate analysis was conducted and statistical significance was defined *p*-value < 0.05. All statistical analyses were performed using the SAS software program (version 9.4).

## Results

### Basic characteristic of participants

The ages of the Young ELEFANT participants in this study were between 24 and 40-years-old (mean age: 29-year-old, SD 4.12). The mean BMI was 22.53 kg/m^2^ (SD 3.85), with 19% of the participants (11,623) classified as overweight and 7% (4252) as obese. Overall, 7% of participants were current alcohol drinkers, 1% were smokers and 25% were exposed to second-hand smoking. The majority of participants maintained a balanced diet and only 2% were on an imbalanced diet. Further, 44% of participants were highly educated (≥16 years of schooling), more than 98% were employed, 24% of the participants experienced at least one of work-related, social or financial stress, and 3% participants were classified as hypertensive. For menarche age, 16% (9347) of the young female participants had menarche at age ≤ 12, 22% at age 13, 39% at age 14, 15% at age 15 and 8% at age ≥ 16 (Table 1).

### Hypertension risk by menarche age and lifestyles

**Fig. 2:**
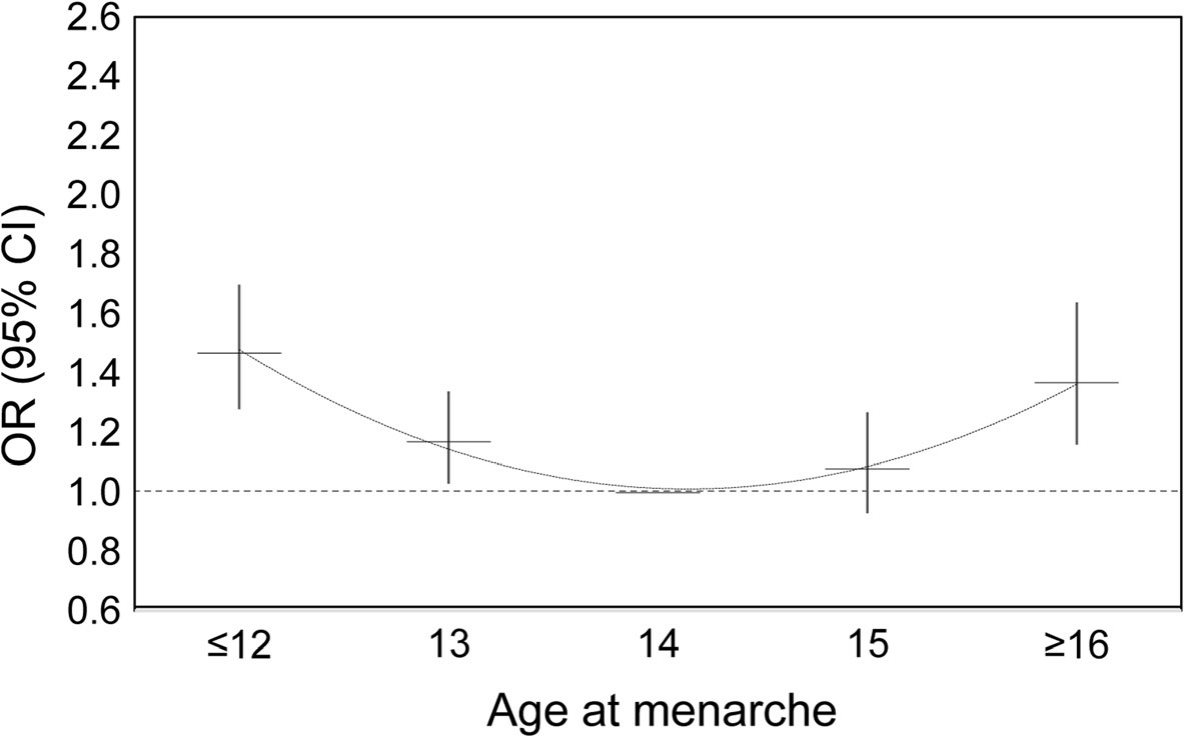
Odds ratios (95% CIs) for hypertension by category of age at menarche among young women. The model was adjusted for age at enrolment, smoking status, passive smoking status, drinking status, imbalanced diet, education, occupation, region, psychological stress, parity, oral contraceptive use, diabetes, and family history of hypertension

To understand the risk of hypertension in young adults, we calculated ORs for hypertension by each age at menarche group. The ORs of hypertension showed a U-shaped distribution, with the highest ORs observed in women with age at menarche of **≤**12 (OR = 1.47, 95% CI: 1.28, 1.70) and **≥** 16 (OR = 1.37, 95% CI: 1.16, 1.64) (Fig. 2 and Additional file 1). The analysis was adjusted for age at enrolment, smoking status, passive smoking status, drinking status, education, occupation, residential region, psychological stress, diabetes, family history of hypertension and reproductive characteristics including parity, and oral contraceptive use.

We next examined whether ORs of hypertension were associated with lifestyles during their young adult life (Table 2). Among young women, higher ORs of hypertension were associated with high BMI (overweight: OR = 2.63, 95% CI: 2.35, 2.96 and obese: OR = 6.50, 95% CI: 5.72, 7.37), psychological stress (OR = 1.38, 95% CI: 1.21, 1.57), passive smoking (OR = 1.55, 95% CI: 1.37, 1.75) and imbalanced diet (OR = 1.25, 95% CI: 1.02, 1.72). Active smoking behaviour was not associated with hypertension (OR = 1.00, 95% CI: 0.70, 1.35). All analyses were adjusted for age at enrolment, drinking status, education, occupation, region, parity, oral contraceptive use, diabetes, family history of hypertension, age at menarche and each other lifestyle.

**Table 2:** Odds ratios (95% CIs) for hypertension among young women by lifestyles

Lifestyle	Participants (*n*)	Hypertension (*n*)	OR	95% CI
BMI				
Normal weight	43,539	739	1.00	Ref
Overweight	11,613	553	2.63	2.35, 2.96
Obesity	4244	506	6.50	5.72, 7.37
Psychological stress				
No	45,829	1122	1.00	Ref
Yes	14,306	676	1.38	1.21, 1.57
Passive smoking				
No	45,243	1081	1.00	Ref
Yes	14,892	717	1.55	1.37, 1.75
Smoke				
No	59,361	1749	1.00	Ref
Yes	774	49	1.00	0.70, 1.35
Imbalanced diet				
No	58,785	1729	1.00	Ref
Yes	1350	69	1.25	1.02, 1.72

Odds ratios were adjusted for age at enrolment, drinking status, education, occupation, region, parity, oral contraceptive use, diabetes, family history of hypertension, age at menarche and each other lifestyle

**Fig. 3:**
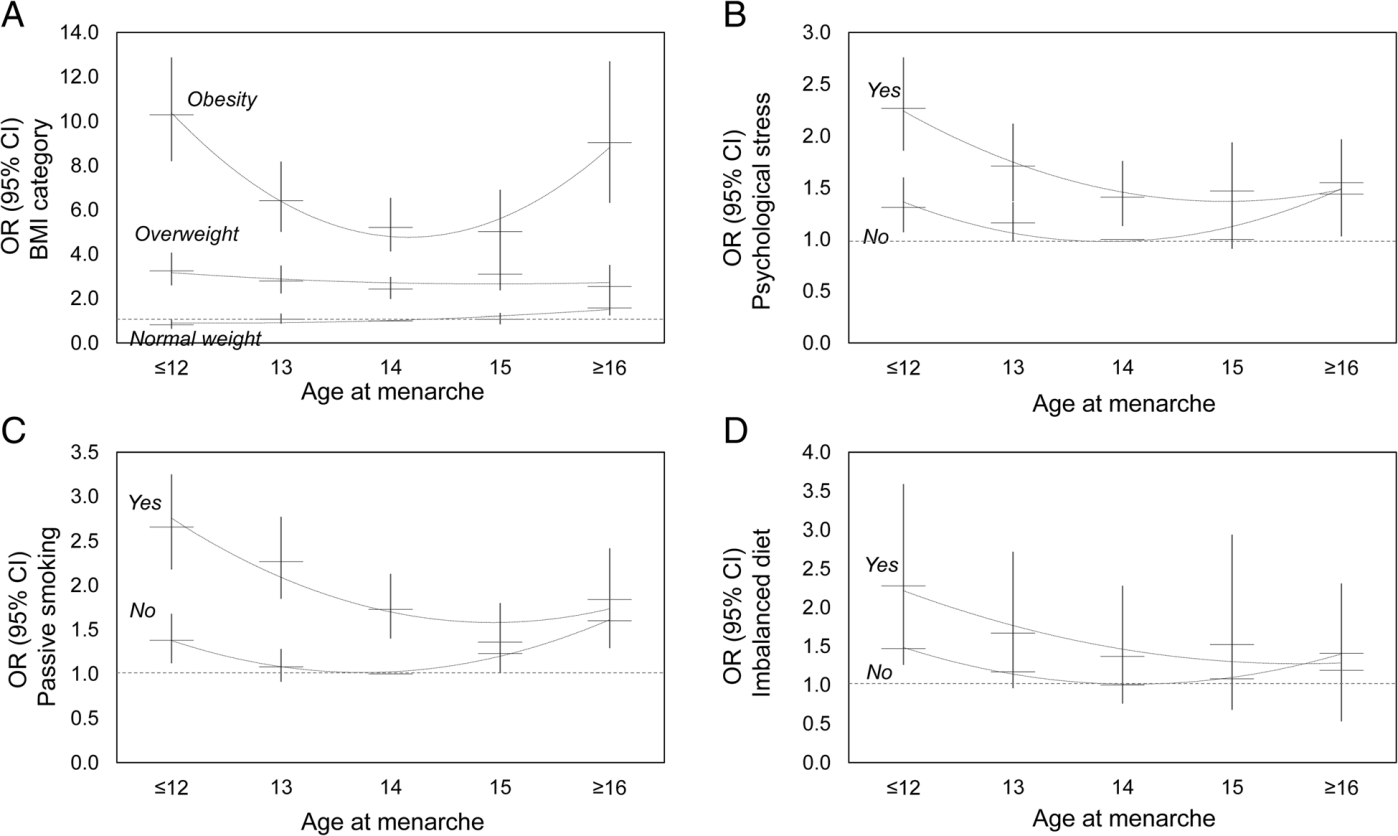
Odds ratios (95% CIs) for hypertension by age at menarche with lifestyles. The analyses were adjusted for age at enrolment, drinking status, education, occupation, region, parity, oral contraceptive use, diabetes, family history of hypertension, age at menarche and each other lifestyle

Joint effects between age at menarche and lifestyles on hypertension revealed that there was interaction between participants with age at menarche ≤12 and being overweight (*P*_*interaction=*_0.005) or obese (*P*_*interaction*_ < 0.001) (Fig. 3 and Additional file 2). There were no interactions between age at menarche with other lifestyles (Fig. 3 and Additional files 3, 4 and 5).

## Hypertension risk by combination of modifiable risk factors with age at menarche

Each lifestyle risk factor independently displayed an association with age at menarche in the risk of hypertension. To examine how the combined associations of lifestyle risk factors and age at menarche may increase the risk of hypertension, we categorised patients by the sum of high-risk lifestyle factors. For example, the lowest risk group were defined as those with healthy weight, no psychological stress, not exposed to passive smoking, and maintained a balance diet, which accounted for 49.46% of the study population. The highest risk group comprised participants who were overweight or obese, reported psychological stress, exposed to passive smoking and had an imbalanced diet, which corresponded to 0.17% of the study population. We observed that late menarche age (≥16) alone was still associated with increased risk of hypertension (OR = 2.07, 95% CI: 1.54, 2.76) in participants with none of the high-risk lifestyles (Table 3). The risk of hypertension increased with greater numbers of high risk lifestyles, particularly among individuals with menarche at age ≤ 12, ranging from 0.53 (95% CI: 0.31, 0.84) in the lowest risk group to 13.21 (95% CI: 5.17, 29.36) in the highest risk group. The risk of hypertension among individuals with menarche age of 14 increased from 1.00 (reference) in the lowest risk group to 7.13 (95% CI: 1.13, 24.8) in the highest risk group (Table 3).

**Table 3:** Odds ratios (95% CIs) for hypertension related to age at menarche by different combinations of modifiable risk lifestyles among young women

Joint Exposure			Hypertension		
Age at menarche (years)	Modifiable risk lifestyles	Total (*n*)	*n*	OR	95% CI
≤12	No risk lifestyles	2747	18	0.53	0.31, 0.84
13		6113	89	1.07	0.83, 1.39
14		13,216	181	1.00	Ref
15		4640	72	1.11	0.84, 1.46
≥16		2139	63	2.07	1.54, 2.76
≤12	One risk lifestyle	2582	90	2.76	2.09, 3.60
13		4001	125	2.25	1.78, 2.85
14		6667	207	2.16	1.76, 2.66
15		2733	98	2.51	1.95, 3.22
≥16		1701	61	2.54	1.87, 3.41
≤12	Two risk lifestyles	2656	156	5.24	4.11, 6.69
13		2347	113	3.98	3.07, 5.14
14		2212	92	3.32	2.54, 4.33
15		1180	48	3.18	2.24, 4.41
≥16		897	47	3.81	2.67, 5.32
≤12	Three risk lifestyles	910	141	12.36	9.51, 16.05
13		580	84	11.09	8.21, 14.87
14		527	59	8.62	6.20, 11.85
15		219	22	7.20	4.35, 11.40
≥16		172	18	7.64	4.39, 12.57
≤12	All four risk lifestyles	40	7	13.21	5.17, 29.36
13		21	3	9.45	2.16, 29.08
14		23	2	7.13	1.13, 24.80
15		5	1	12.23	0.61, 83.43
≥16		9	1	6.49	0.35, 36.53

Modifiable risk lifestyles comprised being overweight or obese, psychological stress, passive smoking, and imbalanced diet. The model was adjusted for age at enrolment, smoking status, drinking status, education, occupation, region, parity, oral contraceptive use, diabetes, and family history of hypertension

## Discussion

Our analysis of a large cohort of young adult Chinese women revealed that both early and late age at menarche are associated with the risk of hypertension. Among lifestyles known to be risk factors for hypertension, BMI showed interaction with age at menarche. To the best of our knowledge, our study is the first to demonstrate that the risk of hypertension in a vulnerable population, that is young adult women with menarche age of **≤**12 or ≥ 16, is substantially increased among women with other key risk factors (higher BMI, psychological stress, passive smoking and an imbalanced diet). This finding suggests that the risk of hypertension in this population could be mitigated through lifestyle modification, and thereby provides huge public health, societal and economic benefit.

High blood pressure increases the risk of many other conditions, including heart disease, heart attacks, strokes, heart failure, kidney disease and vascular dementia. Risk factors for hypertension include age, being overweight or obese, dietary habits (e.g. high consumption of salt, alcohol or coffee and lower intake of vegetables), sedentary lifestyle, and insufficient sleep. In this study, passive smoking was associated with prevalence of hypertension in young adult women, but not with active smoking. For cultural reasons, active smoking may have been underreported in this cohort of young Chinese women, which may therefore serve to explain the apparent absence of an association with hypertension in our study [17]. The risk of hypertension is therefore modifiable by adopting healthy lifestyles, although there are also inherited risk factors, including sex, ethnicity and family history of the disease. It is not yet clear whether those individuals inheriting such risk factors could substantially benefit in reducing their risk of hypertension through lifestyle changes.

Early age at menarche has been shown to be associated with increased risk of hypertension in middle-aged and elderly Western women, and an association between late age at menarche and hypertension or other cardiovascular disease was recently reported in a large UK cohort [7, 18]. In this ELEFANT study, we similarly observed that young Chinese women with either early or late menarche age are at increased risk of developing hypertension. The pathophysiology underlying this association has not been elucidated, but may be related to obesity later in life. Early age at menarche is associated with increased adiposity in adulthood independent of childhood BMI, and especially with increased BMI in adult women below the age of 40 [19]. Our study examined the risk of hypertension among women under 40 and revealed this to be particularly high among obese women with early menarche, thereby identifying a population who could benefit from intervention strategies to reduce CVD incidence. In contrast, as later age at menarche is associated with lower BMI later in life [20], its association with hypertension may be through alternative mechanisms. One such possibility is through reduced exposure to oestrogen, which can reduce blood pressure through stimulation of endothelial nitric oxide synthase [21], although there is conflicting evidence for the effect of oestrogen upon blood pressure [22].

Young adults with even mildly higher blood pressure display increased incidence of heart disease in later life [23]. However, known as a “silent killer”, undiagnosed hypertension is common especially in young adults [24]. Within our cohort, 90% of participants meeting the criteria for hypertension diagnosis had not been previously diagnosed (Additional file 6**)**. Our study has revealed that early and late menarche age are associated with the development of hypertension in young adults. This finding has important implications, as the early detection of hypertension and maintenance of healthy blood pressure in young women with earlier and later menarche age could be critical to decrease the incidence CVD in later life.

Modification of lifestyle has been shown to impact upon lowering blood pressure and has been proposed as a non-pharmacological approach to the prevention and treatment of hypertension [25]. Many studies have focused on modification of a single lifestyle factor for prevention [26, 27], though results of multiple interventions, including dietary intervention with increased physical activity, have been demonstrated to be effective in reducing blood pressure in middle-aged (mean age: 50) men and women [28, 29]. We report that risk of hypertension in vulnerable young adult women, those with either early or late menarche age, increases with the additive associations of multiple high-risk lifestyles. The recommendation of healthy lifestyles to those vulnerable individuals when young could be a highly potent and cost-effective means to reduce the incidence of hypertension and CVD in later life.

Our study utilised a large and well-characterised cohort, with complete information on socio-economic status, lifestyles and reproductive characteristics. All clinical and other questionnaire data measures were carefully standardised and assessed. We rigorously controlled for potential confounders to analyse the relative risk of hypertension. Further, the examination of hypertension in young adult women enabled the elucidation of the associations of menarche age on risk without potential confounding through post-menopausal effects.

Our study contains several potential limitations. The age at menarche was recalled at the time of enrolment, when the participants were young adults. However, it has been shown that the self-reported age at menarche is highly correlated with original age at menarche [30]. Secondly, childhood adiposity, which is considered a risk factor for early pubertal timing [19], was not available in this study. Thirdly, physical activity is potentially suitable as a further modifiable lifestyle factor for investigation in relation to modulation of hypertension risk, but such data was not available within the Young ELEFANT study. Finally, the collected dietary data does not include specific analysis of sodium intake, which is associated with the risk of hypertension. In addition, the classification of obesity by BMI score differs between Asian and Western populations, therefore our findings will need to be validated in a Western cohort. Finally, on account of the observational characteristic of cross-sectional studies, residual confounding by unknown factors might exist.

## Conclusions

In summary, we report that early and late age of menarche are strongly associated with risk of hypertension in young adult Chinese women. Among these individuals, there was an additive association of high-risk lifestyles in increasing the risk of hypertension. Our findings suggest that modification of lifestyle, such as consumption of a balanced diet and maintenance of normal weight could substantially reduce the risk of hypertension in high-risk individuals.

## Additional files

Additional file 1: Odds ratios (95% CIs) for hypertension among young women by age at menarche. (DOCX 26 kb)

Additional file 2: Odds ratios (95% CIs) for hypertension related to age at menarche by BMI. (DOCX 27 kb)

Additional file 3: Odds ratios (95% CIs) for hypertension related to age at menarche by psychological stress. (DOCX 26 kb)

Additional file 4: Odds ratios (95% CIs) for hypertension related to age at menarche by passive smoking. (DOCX 26 kb)

Additional file 5: Odds ratios (95% CIs) for hypertension related to age at menarche by imbalanced diet. (DOCX 27 kb)

Additional file 6: Distribution of age and BMI among undiagnosed hypertension (*n* = 1654) and diagnosed hypertension (*n* = 144) in Young ELEFANT. (DOCX 25 kb)

## Abbreviations

BP: Blood pressure
Cis: confidence intervals
CVD: cardiovascular disease
DBP: diastolic blood pressure
ELEFANT: Environmental and LifEstyle FActors iN metabolic health throughout life-course Trajectories
ORs: odds ratios
SBP: systolic blood pressure
