# Supplementary material for: Age at menarche and prevention of hypertension through lifestyle in young Chinese adult women: result from project ELEFANT
Source: BMC Womens Health. 2018 Nov 9;18:182. doi: 10.1186/s12905-018-0677-y (PMC6234770; doi:10.1186/s12905-018-0677-y)
Supplement: Supplementary file 1 — Odds ratios (95% CIs) for hypertension among young women by age at menarche. (DOCX 26 kb) [file 12905_2018_677_MOESM1_ESM.docx]

**Additional file 1. Odds ratios (95% CIs) for hypertension among young women by age at menarche**

|  |  | **Age at menarche (years)** | | | | |
| --- | --- | --- | --- | --- | --- | --- |
|  |  | **≤12** | **13** | **14** | **15** | **≥16** |
| **No. of participants** |  | 9347 | 13476 | 23186 | 9018 | 5108 |
| **No. of hypertension** |  | 412 | 414 | 541 | 241 | 190 |
| **Multivariable model** | **OR** | 1.47 | 1.17 | 1.00 | 1.08 | 1.37 |
|  | **95% CI** | 1.28, 1.70 | 1.03, 1.34 | **Ref** | 0.93, 1.27 | 1.16, 1.64 |

Odds ratios and 95% CIs were estimated by the logistic regression model adjusted for potential confounding factors. Multivariable model included age at enrolment, smoking status, passive smoking status, drinking status, imbalanced diet, education, occupation, region, psychological stress, diabetes, family history of hypertension and reproductive characteristics including parity, oral contraceptive use for adjustment.
