# Supplementary material for: Age at menarche and prevention of hypertension through lifestyle in young Chinese adult women: result from project ELEFANT
Source: BMC Womens Health. 2018 Nov 9;18:182. doi: 10.1186/s12905-018-0677-y (PMC6234770; doi:10.1186/s12905-018-0677-y)
Supplement: Supplementary file 2 — Odds ratios (95% CIs) for hypertension related to age at menarche by BMI. (DOCX 27 kb) [file 12905_2018_677_MOESM2_ESM.docx]

**Additional file 2. Odds ratios (95% CIs) for hypertension related to age at menarche by BMI**

| **Joint Exposure** | |  | **Hypertension** | |  |
| --- | --- | --- | --- | --- | --- |
| **Age at menarche (years)** | **BMI category** | **Total *n*** | ***n*** | **OR** | **95%CI** |
| ≤12 | Normal weight | 6393 | 102 | 0.83 | 0.64, 1.05 |
| 13 |  | 9851 | 172 | 1.08 | 0.88, 1.31 |
| 14 |  | 17393 | 254 | 1.00 | Ref |
| 15 |  | 6817 | 112 | 1.07 | 0.85, 1.34 |
| ≥16 |  | 3824 | 99 | 1.57 | 1.23, 1.99 |
| ≤12 | Overweight | 2060 | 136 | 3.25 | 2.59, 4.0 |
| 13 |  | 2652 | 129 | 2.79 | 2.23, 3.48 |
| 14 |  | 4319 | 164 | 2.43 | 1.98, 2.97 |
| 15 |  | 1621 | 80 | 3.10 | 2.37, 4.01 |
| ≥16 |  | 961 | 44 | 2.54 | 1.79, 3.51 |
| ≤12 | Obesity | 894 | 174 | 10.30 | 8.23, 12.88 |
| 13 |  | 973 | 113 | 6.42 | 5.01, 8.18 |
| 14 |  | 1474 | 123 | 5.21 | 4.13, 6.55 |
| 15 |  | 580 | 49 | 5.02 | 3.58, 6.91 |
| ≥16 |  | 323 | 47 | 9.05 | 6.32, 12.72 |

Odds ratios were adjusted for age at enrolment, smoking status, passive smoking status, drinking status, imbalanced diet, education, occupation, region, psychological stress, parity, oral contraceptive use, diabetes, and family history of hypertension.
