# Supplementary material for: Age at menarche and prevention of hypertension through lifestyle in young Chinese adult women: result from project ELEFANT
Source: BMC Womens Health. 2018 Nov 9;18:182. doi: 10.1186/s12905-018-0677-y (PMC6234770; doi:10.1186/s12905-018-0677-y)
Supplement: Supplementary file 3 — Odds ratios (95% CIs) for hypertension related to age at menarche by psychological stress. (DOCX 26 kb) [file 12905_2018_677_MOESM3_ESM.docx]

**Additional file 3. Odds ratios** **(95% CIs) for hypertension related to age at menarche by psychological stress**

| **Joint Exposure** | | **Total *n*** | **Hypertension** | |  |
| --- | --- | --- | --- | --- | --- |
| **Age at menarche (years)** | **Psychological stress** |  | ***n*** | **OR** | **95%CI** |
| ≤12 | No | 5050 | 146 | 1.31 | 1.07, 1.60 |
| 13 |  | 9922 | 248 | 1.16 | 0.98, 1.36 |
| 14 |  | 19690 | 412 | 1.00 | Ref |
| 15 |  | 7299 | 176 | 1.00 | 0.91, 1.31 |
| ≥16 |  | 3905 | 141 | 1.55 | 1.27, 1.89 |
| ≤12 | Yes | 4318 | 267 | 2.27 | 1.86, 2.76 |
| 13 |  | 3567 | 167 | 1.71 | 1.37, 2.12 |
| 14 |  | 3511 | 129 | 1.41 | 1.13, 1.76 |
| 15 |  | 1729 | 66 | 1.47 | 1.10, 1.94 |
| ≥16 |  | 1214 | 51 | 1.44 | 1.03, 1.97 |

Odds ratios were adjusted for age at enrolment, smoking status, passive smoking status, drinking status, imbalanced diet, education, occupation, region, parity, oral contraceptive use, diabetes, and family history of hypertension.
