# Supplementary material for: Age at menarche and prevention of hypertension through lifestyle in young Chinese adult women: result from project ELEFANT
Source: BMC Womens Health. 2018 Nov 9;18:182. doi: 10.1186/s12905-018-0677-y (PMC6234770; doi:10.1186/s12905-018-0677-y)
Supplement: Supplementary file 4 — Odds ratios (95% CIs) for hypertension related to age at menarche by passive smoking. (DOCX 26 kb) [file 12905_2018_677_MOESM4_ESM.docx]

**Additional file 4. Odds ratios** **(95% CIs) for hypertension related to age at menarche by passive smoking**

| **Joint Exposure** | | **Total *n*** | **Hypertension** | |  |
| --- | --- | --- | --- | --- | --- |
| **Age at menarche (years)** | **Passive smoking** |  | ***n*** | **OR** | **95%CI** |
| ≤12 | No | 5244 | 156 | 1.38 | 1.12, 1.68 |
| 13 |  | 9854 | 225 | 1.08 | 0.91, 1.28 |
| 14 |  | 19503 | 395 | 1.00 | Ref |
| 15 |  | 7092 | 180 | 1.23 | 1.02, 1.47 |
| ≥16 |  | 3550 | 125 | 1.60 | 1.29, 1.97 |
| ≤12 | Yes | 4103 | 256 | 2.66 | 2.18, 3.25 |
| 13 |  | 3622 | 189 | 2.27 | 1.85, 2.77 |
| 14 |  | 3683 | 146 | 1.73 | 1.40, 2.13 |
| 15 |  | 1926 | 61 | 1.36 | 1.01, 1.80 |
| ≥16 |  | 1558 | 65 | 1.84 | 1.38, 2.42 |

Odds ratios were adjusted for age at enrolment, smoking status, drinking status, imbalanced diet, education, occupation, region, psychological stress, parity, oral contraceptive use, diabetes, and family history of hypertension.
