# Supplementary material for: Age at menarche and prevention of hypertension through lifestyle in young Chinese adult women: result from project ELEFANT
Source: BMC Womens Health. 2018 Nov 9;18:182. doi: 10.1186/s12905-018-0677-y (PMC6234770; doi:10.1186/s12905-018-0677-y)
Supplement: Supplementary file 5 — Odds ratios (95% CIs) for hypertension related to age at menarche by imbalanced diet. (DOCX 27 kb) [file 12905_2018_677_MOESM5_ESM.docx]

**Additional file 5. Odds ratios** **(95% CIs) for hypertension related to age at menarche by imbalanced diet**

| **Joint Exposure** | | **Total *n*** | **Hypertension** | |  |
| --- | --- | --- | --- | --- | --- |
| **Age at menarche (years)** | **Imbalanced diet** |  | ***n*** | **OR** | **95%CI** |
| ≤12 | No | 5252 | 157 | 1.47 | 1.26, 1.70 |
| 13 |  | 9860 | 225 | 1.17 | 1.02, 1.34 |
| 14 |  | 19511 | 395 | 1.00 | Ref |
| 15 |  | 7097 | 180 | 1.08 | 0.92, 1.27 |
| ≥16 |  | 3556 | 125 | 1.41 | 1.18, 1.68 |
| ≤12 | Yes | 4116 | 256 | 2.28 | 1.37, 3.59 |
| 13 |  | 3629 | 190 | 1.67 | 0.96, 2.72 |
| 14 |  | 3690 | 146 | 1.37 | 0.76, 2.28 |
| 15 |  | 1931 | 62 | 1.52 | 0.68, 2.94 |
| ≥16 |  | 1563 | 67 | 1.19 | 0.53, 2.31 |

Odds ratios were adjusted for age at enrolment, smoking status, passive smoking status, drinking status, education, occupation, region, psychological stress, parity, oral contraceptive use, diabetes, and family history of hypertension.
