# Supplementary material for: Age at menarche and prevention of hypertension through lifestyle in young Chinese adult women: result from project ELEFANT
Source: BMC Womens Health. 2018 Nov 9;18:182. doi: 10.1186/s12905-018-0677-y (PMC6234770; doi:10.1186/s12905-018-0677-y)
Supplement: Supplementary file 6 — Distribution of age and BMI among undiagnosed hypertension (n = 1654) and diagnosed hypertension (n = 144) in Young ELEFANT. (DOCX 25 kb) [file 12905_2018_677_MOESM6_ESM.docx]

**Additional file 6.** Distribution of age and BMI among undiagnosed hypertension (*n*=1,654) and diagnosed hypertension (*n*=144) in Young ELEFANT

|  | Mean | Standard deviation | Median | 25%Q1 | 75%Q3 |
| --- | --- | --- | --- | --- | --- |
| Undiagnosed hypertension | | |  |  |  |
| Age (years) | 30.81 | 4.41 | 31.00 | 28.00 | 34.00 |
| BMI (kg/m^2^) | 25.56 | 4.79 | 24.91 | 22.04 | 28.48 |
| Diagnosed hypertension | | |  |  |  |
| Age (years) | 32.40 | 3.49 | 32.50 | 30.00 | 35.00 |
| BMI (kg/m^2^) | 27.04 | 4.46 | 27.10 | 23.74 | 29.55 |
